# Can we adequately teach ethics and ethical decision making via distant learning? A pandemic pilot

**DOI:** 10.3205/zma001373

**Published:** 2020-12-03

**Authors:** Robert Gintrowicz, Klemens Pawloy, Julius Richter, Antje Degel

**Affiliations:** 1 Charité Universitätsmedizin Berlin, Prodekanat für Studium und Lehre, Berlin, Germany; 2 Charité Universitätsmedizin Berlin, Med. Klinik für Kardiologie, Campus Benjamin Franklin, Berlin, Germany

**Keywords:** ethics course, ethical decision making, emergency medicine, distant learning, pandemic, corona virus, undergraduate medical education

## Abstract

The Corona virus pandemic rendered most live education this spring term impossible. Other formats and new ideas were needed to offer students the opportunity to learn unchanged learning content and outcomes.

To replace our module on ethics and ethical decision making in emergency medicine with simulation patients we developed an e-learning module consisting of a case, trigger questions and literature for self-study. This was followed by a Microsoft Teams seminar in which the students discussed their questions in subgroups on the basis of their reading and developed a team product they then presented to the other team.

Students valued this module as enabling a safe space for their beliefs and views. A vast majority deemed the topics as relevant, two thirds would retake the seminar. Despite a productive online discourse, this format should not be used as sole module under normal conditions since it lacks the (simulation) patient interaction but it can prove to be a valuable addendum to live teaching.

## Zusammenfassung

Die Coronavirus-Pandemie machte in diesem Frühjahrssemester die meisten Lehrveranstaltungen in Präsenz unmöglich. Es waren andere Formate und neue Ideen erforderlich, um den Studierenden die Möglichkeit zu bieten, die unveränderten Lerninhalte und -ziele zu erlernen.

Um unser Modul mit Simulationspatienten über Ethik und ethische Entscheidungsfindung in der Notfallmedizin zu ersetzen, entwickelten wir ein E-Learning-Modul, das aus einem Fall, Triggerfragen und Literatur zum Selbststudium besteht. Daran schloss sich ein Microsoft-Teams-Seminar an, in dem die Studierenden ihre Fragen in Untergruppen auf der Grundlage ihrer Lektüre diskutierten und ein Teamprodukt entwickelten, das sie dann dem anderen Team vorstellten.

Die Studenten schätzten dieses Modul als einen sicheren Raum für ihre Überzeugungen und Ansichten. Eine große Mehrheit hielt die Themen für relevant, zwei Drittel würden das Seminar wiederholen. Trotz eines produktiven Online-Diskurses sollte dieses Format unter normalen Bedingungen nicht als alleiniges Modul verwendet werden, da ihm die (Simulations-)Patienteninteraktion fehlt, es kann sich aber als wertvolle Ergänzung zum Live-Unterricht erweisen.

## Introduction

Starting December 2019 in China the novel corona virus SARS-CoV-2 has spread around the world infecting millions and killing thousands [1], [2], [3]. It reached Berlin at the beginning of March [4], and was followed by the prohibition of group aggregations until only household members were allowed to undercut minimal social distance [5]. The beginning of the semester was postponed for all students including medical students at Charité Universitätsmedizin Berlin [6]. Ethics and ethical decision making in emergency situations had been taught with simulation patients or relatives and small group discussions embedded in the emergency medicine course in the tenth semester. We now had to find a different format. First attempts at online ethics teaching have shown non-inferiority in an ethics course combined of lectures and tutorials [7] and a positive effect on student confidence in decision making using an online platform [8].

## Project outline

Whereas the COVID-19 pandemic task force allowed life support simulations for semesters 1, 6 and 10 to be performed in a comprised fashion under strict hygienic standards, our module on ethics and ethical decision making in emergency medicine (the last ethics module prior to their practical year) using simulation patients in semester 10 was cancelled. This was due to the prime directive of protecting (high) risk patients, a group most of our simulation patients belong to (due to age, concurrent diseases etc.). We therefore needed to find an alternative to address these issues with our students that would be ready on time, since the importance of ethics and professionalism in medical education [9] should not be undermined by reducing it to self-study due to the pandemic. Based on the online ethics course in nursing [7] and the reported positive effects of team-based learning on medical ethics education [10] we decided to synthesize both proposals into one online module.

## Summary of work

We developed an online module consisting of online resources offered on our learning platform and a Microsoft Teams live seminar of 90 minutes duration. The online resources comprised two versions of a resuscitation case – differing only in the attached ethical questions – and matching literature citations. One week prior to the online seminar, the 20 to 24 students were informed as to their randomly determined subgroup allocation and asked to read the provided papers, inviting them to conduct further literature research if wanted. Each group had to work on one of the two questions – one pertaining to the presence of relatives during resuscitation and the other to the termination of resuscitative efforts.

In the online seminar, as an activating tool or teaser, we started with a Microsoft Forms survey asking about their prior experience with resuscitation if any, their role therein, and general attitude towards ethics in medicine (see attachment 1 ). Our introductory survey revealed that 72% of our 156 students had not witnessed or assisted a resuscitation effort but 28% had. Most had seen advanced directives but only half of them knew the term “DNAR” (do not attempt resuscitation). An organ donor ID card is carried by 84% of them. Only 53% reported having witnessed an ethical dilemma during their studies or in their life. The results were then discussed with the students sensitizing the group to everyday ethical dilemma they had inadvertently overlooked. 

**Figure 1:**
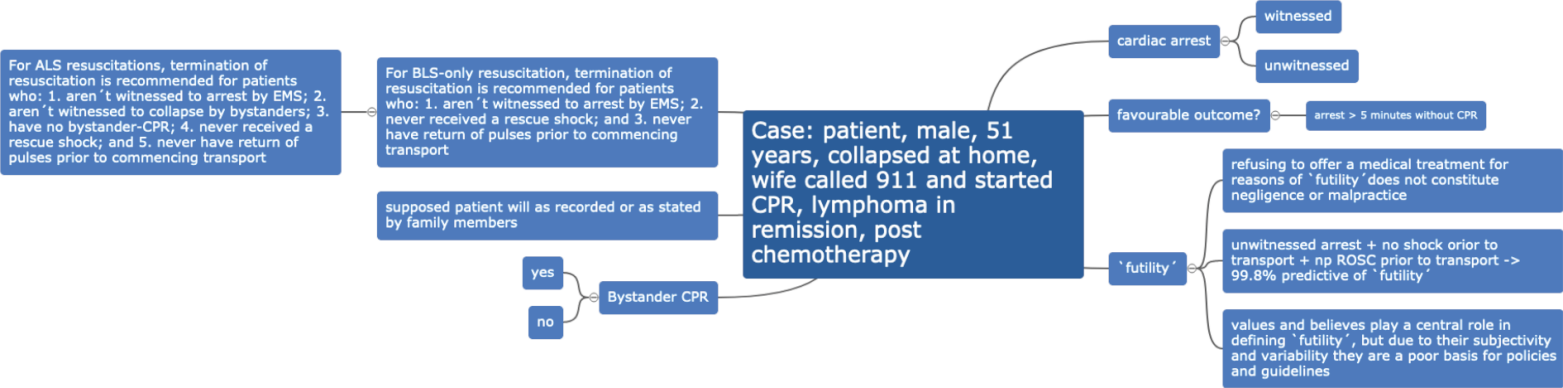
Example of one group product after team-based discussions as presented in the joint closing session

Thereafter, the rules of conduct were set (safe space, no interruptions, the possibility of differing opinions etc.) and the subgroups were guided into private channels attended by tutors (peer teachers or clinical teachers). Here they worked on their question by discussing the literature, presenting further reading and disclosing their feelings and beliefs. Each group created a team product according to their wishes, predilections or abilities as result (see figure 1 (Fig. 1)). These were then presented to the other group and discussed in a joint closing session. 

At the end, we conducted another Forms survey asking for their opinions on the seminar and the concurrent emergency medicine course (see attachment 2 ). 

Albeit having a reduced number of simulations, 85% of our students rated the emergency course as excellent or good. Pertaining to this seminar, 94% viewed it and its contents as relevant for their education, 68% would recommend it to others.

## Discussion

The online module was readily embraced by our students. Apart from minor technical problems joining the subgroup channels no major technical setbacks were recorded. We noticed that – similar to our live sessions – the allocated time is a challenge, online work did not reduce this dilemma. Students wished a preset allocation of literature reading to facilitate preparation of the live online session. Our teachers and participants noted that the online format facilitated open discourse and disclosure of feelings and beliefs. Our teachers and student teachers valued the openness of the discussion and the creativity in producing their presentation. The students seem to be aware of the relevance of the topic, but only 68% said they would recommend it to others. What is missed is the interaction with the simulation patient and their subjective feedback, which cannot be replaced by online seminars [11], [12], [13]. Virtual patients have been tested for narrowly defined settings, and seem to be an effective addendum to standardized patients but no complete surrogate as interaction is limited [14]. We need to improve the acceptance of the online module possibly blending it with live sessions using simulated patients or relatives in the future, or develop a completely new concept of live online interaction with simulation patients, as the trends in telemedicine would command.

## Conclusion

This online module on ethics and ethical decision making in emergency medicine settings is a possibility to sensitize students to the ethical dilemmas that prevail in this context. It offers a safe space for them to open themselves and present their emotions and beliefs without judgement. It cannot, however, completely compensate for the complex doctor-patient interaction that is emulated with simulation patients. Under the given circumstances, it proved feasible and with improvement in acceptance could be a helpful addendum to the existing module in the future (when live simulation patient modules are allowed again). Research into the possibilities of online integration of simulation patients has to be undertaken to be able to evaluate its use in ethical education in undergraduate medicine.

## Competing interests

The authors declare that they have no competing interests. 

## Supplementary Material

Teaser questionnaire at the begin of the session

Closing questionnaire, administered at the end of the session
