# Supplementary figures and images for: Can we adequately teach ethics and ethical decision making via distant learning? A pandemic pilot
Source: GMS J Med Educ. 2020 Dec 3;37(7):Doc80. doi: 10.3205/zma001373 (PMC7740007; doi:10.3205/zma001373)

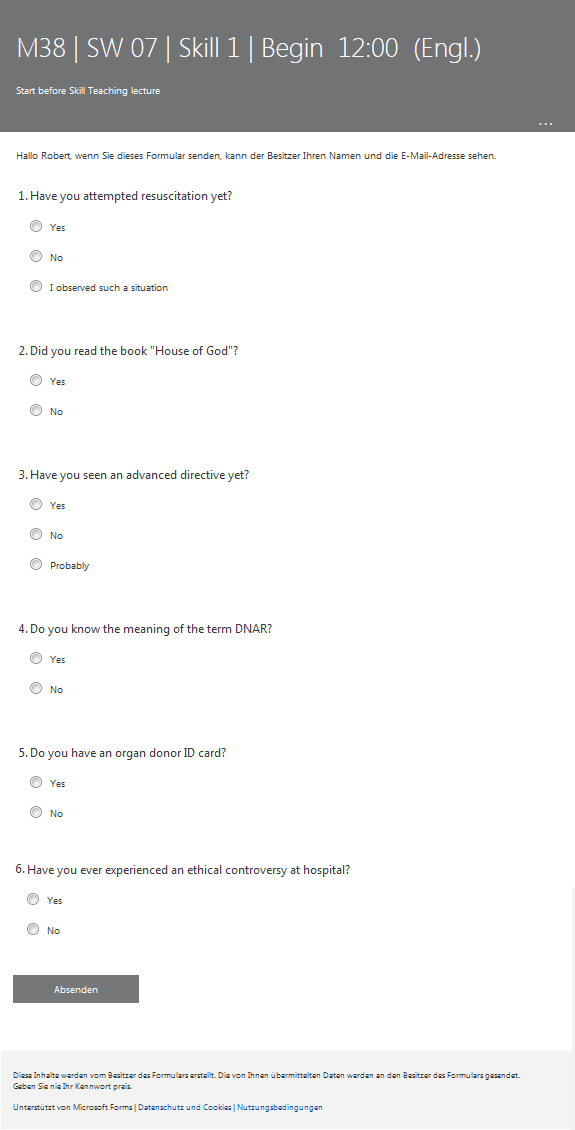

Supplement: Teaser questionnaire at the begin of the session [file JME-37-80-s-001.png]

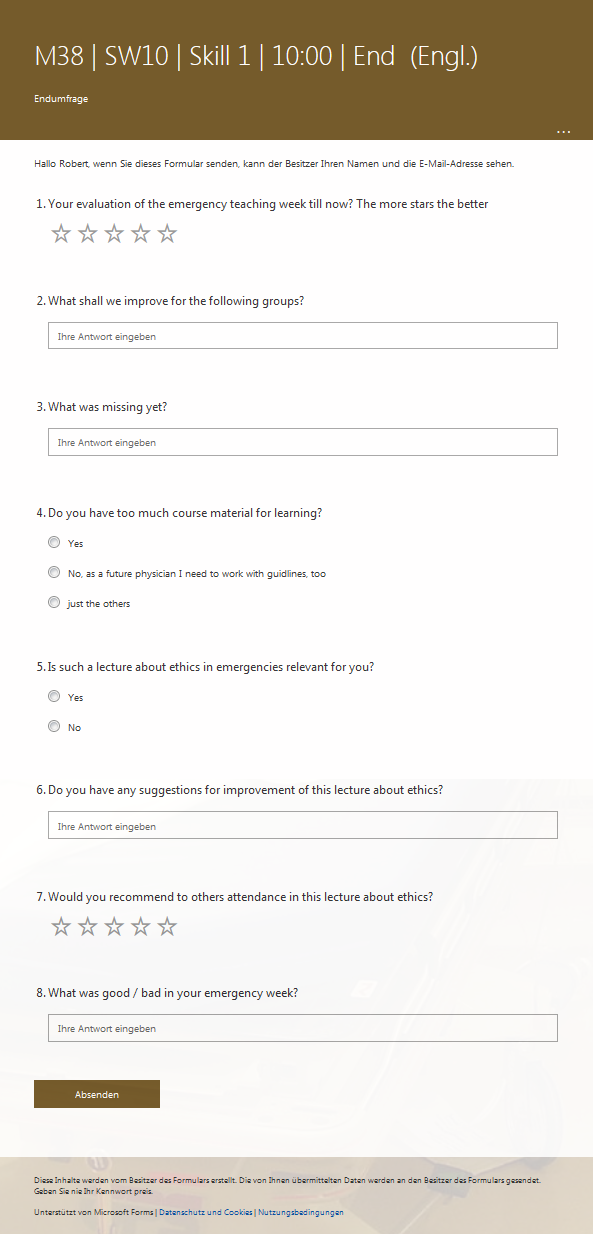

Supplement: Closing questionnaire, administered at the end of the session [file JME-37-80-s-002.png]
